# The Modified Triple Stapler Technique for Colorectal and Coloanal Anastomosis: A Retrospective Study of 132 Patients

**DOI:** 10.7759/cureus.83496

**Published:** 2025-05-05

**Authors:** Ahmed Abdelkader, Abdulsalam Akinlade, Aashir Luqhman, Georgy Thomas, Waleed Naveed, Cyrilkumaar Vijayakumar, Anupam Chandran, Mubariz Mahmoud, Muzaffar Ahmad

**Affiliations:** 1 Surgery, Northern Lincolnshire and Goole NHS Foundation Trust (NLaG), Hull, GBR; 2 General Surgery, Scunthorpe General Hospital, Scunthorpe, GBR; 3 Surgery, Northern Lincolnshire and Goole, Scunthorpe, GBR; 4 Surgery Department, Northern Lincolnshire and Goole NHS Trust (NLaG), Scunthorpe, GBR

**Keywords:** anastomosis leak, colo-anal anastomosis, colo rectal cancer, resection anastomosis, reversal of hartmann's procedure

## Abstract

Aim

The objective of this study was to evaluate the safety and efficacy of the modified triple-staple technique in anterior resection for colorectal cancer patients and Hartmann’s reversal surgeries done by a single surgeon. We hypothesized that by using this technique and omitting the purse-string suture, we could reduce operative time and enhance ergonomic benefits.

The study was conducted in accordance with local clinical governance unit protocols and adhered to the Strengthening the Reporting of Cohort Studies in Surgery (STROCSS) guidelines.

Methods

This retrospective cohort study aimed to evaluate patients who underwent colorectal anastomosis as part of either Hartmann’s reversal or colorectal cancer surgeries over a 15-year period, from January 2010 to January 2025.

A total of 132 patients who underwent anterior resection for colorectal cancer or Hartmann’s reversal were included. A modified triple staple technique for end-to-end colorectal and coloanal anastomosis was performed without the use of a purse-string suture on both the proximal and distal segments. The safety and efficacy of this technique were assessed by monitoring complication rates, including intraoperative complications, anastomotic leaks, and strictures.

Results

There were no major intraoperative complications reported. Anastomotic leakage occurred in 11 patients (8%), while strictures developed in 2 patients (2%). Postoperative abdominal collections were observed in three patients (2%), and postoperative ileus occurred in nine patients (7%).

Conclusion

The modified triple stapler technique for end-to-end anastomosis offers a safe and effective alternative to the conventional purse-string double stapler technique.

## Introduction

This study builds on our previous work in the surgical department at Scunthorpe General Hospital. In 2007, we published a study introducing a novel surgical approach for coloanal anastomosis using a modified triple stapler technique [1]. Our findings demonstrated that this technique produced results comparable to the standard purse-string methods, with the added benefit of enhanced ergonomic advantages.

Anastomotic leakage (AL) is a common and concerning complication faced by colorectal surgeons. It is associated with higher morbidity and mortality rates, as well as negative effects on functional and oncologic outcomes. Additionally, AL places a significant strain on hospital resources [2].

Leak rates in colorectal surgery vary depending on the anatomic location of the anastomosis, with distal colorectal, coloanal, and ileoanal anastomoses exhibiting leak rates ranging from 1% to 20% [3-6]. These variations often lead to differing outcomes in terms of morbidity and mortality [7-8].

Several risk factors contribute to AL, which can be classified into patient-specific, intraoperative, and low rectal anastomosis-specific factors. The latter is particularly associated with an increased risk due to poor colonic vascularity and limited tissue support at the anastomotic site. Patient-specific factors include malnutrition, steroid use, tobacco use, leukocytosis, cardiovascular disease, alcohol consumption, and a higher American Society of Anesthesiologists (ASA) score [9]. Additionally, surgical technique and the surgeon's expertise play significant roles in influencing leakage rates [7-9].

Various modifications to stapling techniques have been developed since their introduction. The double-staple technique remains the most commonly used method, enabling colorectal anastomosis at lower levels and reducing the risk of fecal contamination [10-11].

The triple stapler technique, which involves stapling both the proximal colon and the distal rectal stump, offers the potential for a faster, purse-string-avoiding, and more secure colorectal and coloanal anastomosis. This study aims to evaluate the safety and efficacy of the triple-staple technique performed by a single surgeon in anterior resection and Hartmann’s reversal surgeries.

## Materials and methods

Participants, interventions, comparisons, and outcomes (PICO) research question

Could the modified triple stapler technique offer a better alternative for colorectal and coloanal anastomosis?

Study design and patient selection

We conducted a retrospective cross-sectional study following a predefined protocol recommended by the local clinical governance unit. The study was reported in accordance with the Strengthening the Reporting of Cohort Studies in Surgery (STROCSS) guidelines for observational research. Since the study was retrospective and utilized anonymized data, research ethics committee approval and patient consent were not required.

The study was carried out in the general surgery department at Scunthorpe General Hospital, United Kingdom, and included a sample population of 132 patients over 15 years who underwent either low anterior resection or Hartmann's reversal surgeries. All procedures were performed by a single surgeon using the modified triple stapler technique for anastomosis. The study focused on intraoperative complications, postoperative AL rates, and strictures.

The inclusion criteria for our study included all adult patients, regardless of age or gender, who underwent elective low anterior resections or Hartmann's reversal surgeries with planned primary colorectal or coloanal anastomosis. We excluded emergency operations.

Data collection

A standardized proforma was developed for electronic data collection to ensure comprehensive documentation of key variables, including age, gender, ASA score, type of operation performed, intraoperative complications, mean operative time, presence of a covering ileostomy, postoperative leakage, and the development of strictures. All patients underwent postoperative CT scans when clinically indicated or during routine follow-up and were assessed for complications during face-to-face follow-up appointments.

Operative techniques

During either a laparoscopic or open surgical approach, standard mobilization of the descending colon and rectum is typically performed. Following this, distal transection is carried out, and the segment planned for resection is exteriorized. Once the proximal transection site is identified, a colotomy is created approximately 2-3 cm distal to this point to allow introduction of the anvil, which is then milked proximally with the head positioned cephalad.

**Figure 1:**
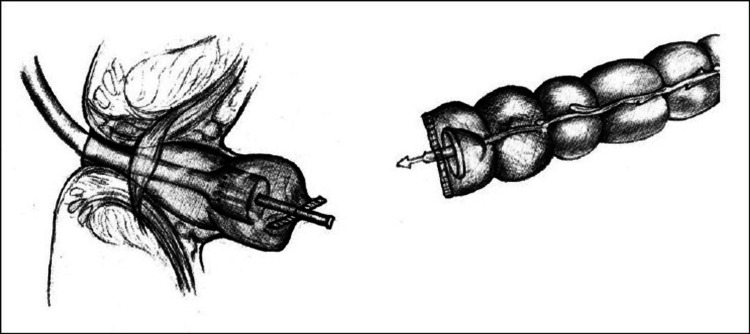
This figure demonstrates the staple line over the distal colonic end and the anvil protruding. Figure sourced from [12], which allows unrestricted reuse.

A GIA linear cutting stapler is subsequently used to divide the bowel, leaving the anvil in situ and freeing the resection specimen. The spike attached to the shaft of the anvil is then advanced through the bowel wall, adjacent to the linear staple line, in preparation for an end-to-end anastomosis (Figure 1).

In cases where there is a discrepancy in diameter between the proximal and distal ends, the anvil may be introduced approximately 5 cm proximally through the antimesenteric taenia to facilitate a side-to-end anastomosis. However, this approach carries potential disadvantages, including a reduced blood supply to the distal stump and increased difficulty in performing endoscopic surveillance of the stump in the future.

## Results

A total of 132 patients were included in the study, comprising 95 who underwent anterior resection and 37 who underwent Hartmann’s reversal between January 2010 and January 2025.

Demographic data are summarized in Table 1. The mean patient age was 64.7 years (range: 45-87 years), with a median age of 64. Males accounted for 51% (n=68) of the cohort, while females made up 49% (n=64). The ASA (American Society of Anesthesiologists) classification was also assessed, with the majority of patients falling into ASA II and ASA III categories, indicating the presence of mild to moderate systemic disease.

**Table 1:** Demographics The data has been represented as a number and a percentage. LOS: length of hospital stay; OT: operative time

Number of Cases	132
Age (years)	(45-87)
Mean	66
Median	64.5
Sex	
Male	68 (51%)
Female	64 (49%)
ASA score	
1	8 (6%)
2	52 (39%)
3	62 (47%)
Indication for surgery	
Anterior resection	95 (72%)
Hartmann's reversal	37 (28%)
covering ileostomies	95 (72%)
mean OT (mins)	211
mean LOS (days)	9.5
Complications	
Anastomotic leaks	11 (8%)
Anastomotic strictures	3 (2%)
Postoperative ileus	9 (7%)
Postoperative collections	2 (2%)
Major intraoperative complications	none

The outcomes demonstrated no major intraoperative complications. The AL rate was 8% (11 patients), while the stricture rate was 2% (3 patients). Postoperative abdominal collections occurred in 2% (2 patients), and 7% (9 patients) developed postoperative ileus (Table 2).

**Table 2:** Complications The data was presented as a number and a percentage.

Complications	
Anastomotic leaks	11 (8%)
Anastomotic strictures	3 (2%)
Postoperative ileus	9 (7%)
Postoperative collections	2 (2%)
Major intraoperative complications	None

## Discussion

AL remains the most critical and feared complication following colorectal resections. It not only contributes to increased postoperative morbidity and prolonged hospital stays but is also associated with higher rates of reoperation, poor functional outcomes, and mortality. As such, it is commonly considered a primary indicator of success in rectal anastomosis. Given these risks, ongoing efforts have been made to refine anastomotic techniques in order to reduce the incidence of leaks, streamline surgical workflows, and improve outcomes.

The laparoscopic approach has gained widespread acceptance as the standard of care for a majority of colorectal procedures. It offers well-documented advantages, including reduced postoperative pain, faster recovery, shorter hospital stays, and fewer wound-related complications. Importantly, studies have shown that these benefits do not come at the expense of oncologic integrity, making laparoscopy a compelling option even for cancer resections [13].

Despite these advances, creating a secure and reliable anastomosis, particularly in the lower rectum, remains technically challenging. The difficulty increases in patients with poor pelvic exposure, such as those who are obese, male, or have bulky distal rectal tumors. These anatomical constraints can complicate visibility and maneuverability, increasing the technical demand on the surgeon during critical steps such as anvil placement and staple firing.

Traditionally, the double-staple technique has been the cornerstone method for colorectal anastomosis following anterior resection. It involves the creation of a rectal stump using a linear stapler, followed by the insertion of a circular stapler anvil into the proximal colon. A purse-string suture is required to secure the anvil in place, after which the circular stapler is introduced trans-anally to complete the anastomosis [14].

However, this technique is not without limitations. One major concern is the potential for fecal contamination during the creation of the purse-string suture. Opening the bowel lumen to insert the anvil increases the risk of intraperitoneal soiling, which may contribute to pelvic sepsis and subsequent complications. Additionally, in patients with proximal bowel dilation due to obstruction, achieving an effective purse-string closure becomes difficult. The mismatch in bowel diameters may lead to an incomplete seal, further jeopardizing anastomotic integrity [8,10,15].

The modified triple stapler technique has emerged as a potential solution to these issues. By using a linear stapler to close and transect the proximal colon, the bowel remains sealed, minimizing the risk of contamination. This approach eliminates the need for a purse-string suture. Moreover, in cases with significant size discrepancy between the proximal and distal bowel ends, a side-to-end anastomosis can be created more easily, enhancing adaptability while maintaining the principles of a tension-free, well-perfused connection.

A common concern with the triple stapler technique involves the crossing of staple lines and the theoretical risk of impaired healing or reduced anastomotic blood flow. However, several experimental studies in animal models have investigated this issue. In canine studies, linear staples intersecting at the anastomotic site were observed to be deformed, cut, or compressed within the excised tissue doughnuts, indicating that the presence of intersecting staple lines does not necessarily compromise anastomotic integrity [16].

Further support comes from both experimental and clinical studies demonstrating that intersecting staple lines do not significantly reduce perfusion at the anastomotic site. These studies found no meaningful difference in leakage rates or healing outcomes when staple lines overlapped, suggesting that concerns about vascular compromise may be overstated [8,17]. Additionally, real-world surgical experience has shown that stapling across a staple line, when performed under controlled conditions, is both safe and effective.

The ergonomic benefits of the triple stapler technique also warrant attention. In the narrow confines of the pelvis, particularly in laparoscopic settings, simplifying anastomotic construction by eliminating purse-string placement can significantly reduce operative complexity. This can lead to shorter operative times and potentially lower surgeon fatigue, especially during prolonged procedures or in technically demanding cases.

Although this was not a comparative study, the outcomes observed were consistent with and comparable to those reported in the existing literature. Despite the absence of a direct control group, key indicators such as anastomotic leak rate, postoperative complications, and overall morbidity fell within the expected range of established benchmarks from previous studies evaluating conventional stapling techniques. This alignment with published data supports the feasibility and safety of the modified triple stapler technique and suggests that it may offer outcomes equivalent to standard methods currently in use. However, formal comparative studies are required to validate these findings and determine whether this approach provides any significant advantages over traditional techniques [18].

Overall, the modified triple stapler technique presents a promising evolution in colorectal surgery. It offers practical advantages in terms of safety, efficiency, and adaptability, particularly in anatomically challenging patients. However, its widespread adoption will depend on further evidence from well-designed comparative studies. Prospective trials and long-term follow-up data are essential to determine whether the observed short-term benefits translate into superior outcomes and reduced complication rates.

Limitations of the study

This study has several limitations. Notably, the absence of a control or comparative group restricts the ability to draw definitive conclusions about the relative effectiveness of the modified triple stapler technique when compared to other established anastomotic methods. Without direct comparison, it is difficult to determine whether the observed outcomes are attributable to the technique itself or to other factors such as patient selection, surgical expertise, or perioperative management protocols. As a result, while the findings are encouraging, they should be interpreted with caution until validated through prospective, controlled studies.

## Conclusions

The modified triple stapler technique for end-to-end colorectal and coloanal anastomosis offers notable ergonomic advantages for the operating surgeon, particularly in challenging cases where discrepancy is present between the proximal and distal ends. By simplifying the alignment and stapling process, this technique enhances surgical precision and may contribute to reduced operative time and fatigue. Clinical outcomes associated with this approach demonstrate safety and efficacy comparable to conventional stapling methods. As such, the modified triple stapler technique represents a promising and practical alternative to traditional techniques. With further validation and training, it has the potential to be widely adopted in routine clinical practice, particularly in procedures such as anterior resections and Hartmann's reversal, where ease of anastomosis and reliable outcomes are paramount.
